# Acoustic stimulation of the human round window by laser-induced nonlinear optoacoustics

**DOI:** 10.1038/s41598-024-58129-0

**Published:** 2024-04-08

**Authors:** Liza Lengert, Michael Tomanek, Mohammad Ghoncheh, Hinnerk Lohmann, Nils Prenzler, Stefan Kalies, Sonja Johannsmeier, Tammo Ripken, Alexander Heisterkamp, Hannes Maier

**Affiliations:** 1 Laser Zentrum Hannover E.V., Hollerithallee 8, 30419 Hannover, Germany; 2 Department of Otorhinolaryngology and Cluster of Excellence “Hearing4all”, Hannover Medical School, VIANNA/NIFE, Stadtfelddamm 34, 30625 Hannover, Germany; 3 Institute of Quantum Optics, Leibniz University Hannover, Hannover, Germany; 4 NIFE, Lower Saxony Center for Biomedical Engineering, Implant Research and Development, Hannover, Germany

**Keywords:** Electro-acoustic stimulation, Active middle ear implant, Optoacoustic, Inner ear, Photoacoustics

## Abstract

The feasibility of low frequency pure tone generation in the inner ear by laser-induced nonlinear optoacoustic effect at the round window was demonstrated in three human cadaveric temporal bones (TB) using an integral pulse density modulation (IPDM). Nanosecond laser pulses with a wavelength in the near-infrared (NIR) region were delivered to the round window niche by an optical fiber with two spherical lenses glued to the end and a viscous gel at the site of the laser focus. Using IPDM, acoustic tones with frequencies between 20 Hz and 1 kHz were generated in the inner ear. The sound pressures in scala tympani and vestibuli were recorded and the intracochlear pressure difference (ICPD) was used to calculate the equivalent sound pressure level (eq. dB SPL) as an equivalent for perceived loudness. The results demonstrate that the optoacoustic effect produced sound pressure levels ranging from 140 eq. dB SPL at low frequencies ≤ 200 Hz to 90 eq. dB SPL at 1 kHz. Therefore, the produced sound pressure level is potentially sufficient for patients requiring acoustic low frequency stimulation. Hence, the presented method offers a potentially viable solution in the future to provide the acoustic stimulus component in combined electro-acoustic stimulation with a cochlear implant.

## Introduction

The World Health Organization (WHO) estimates that over 430 million people, > 5% of the world’s population, currently suffer from disabling hearing loss^1^. In profound deaf patients, cochlea implants (CI) are a widely used method for restoring the patient’s ability to hear by electrically stimulating the spiral ganglion cells in the inner ear. These implants can restore speech recognition in adults and provide even better results in children. However, CIs have major technical limitations that lead to insufficient speech intelligibility in noisy environments and/or music perception. Mainly, the achievable frequency resolution of these implants is limited by the spread of the current to surrounding cells^2–4^. To overcome some of these limitations other methods of stimulation need to be considered. One clinically successful approach is the use of acoustic stimulation at frequencies < 1.5 kHz in combination with a CI for higher frequencies to improve speech intelligibility in noisy environments. However, besides some residual hearing at low frequencies this combined electro-acoustic stimulation (EAS) requires an intact middle ear and thus is not an option for people suffering from conductive hearing loss^5^.

Alternative concepts are needed to improve the frequency resolution. A promising approach is the stimulation by optical means e.g. through optogenetics^6,7^. In vivo experiments have been successfully performed using optical CIs consisting of a string of small LEDs to perform an optical stimulation of genetically modified spiral ganglion neurons (SGNs) while providing a better spatial resolution than electrical CIs^7^. Besides the need to modify the spiral ganglion cells genetically before stimulation, one main drawback of optogenetic stimulation is a low temporal fidelity of most light sensitive channels. A recent study by Keppeler et al. suggests however, that the temporal fidelity can be vastly improved by using the Chronos ES/TS light-sensitive protein^8^.

Another optical stimulation method utilizes near-infrared (NIR) laser pulses to directly stimulate neurons such as cortical neurons or the sciatic nerve^2,9–11^. However, Kallweit et al. showed that the stimulation of the cochlea via NIR laser radiation is not directly caused by neuronal stimulation but by the optoacoustic effect, which evokes a pressure wave and consequently an activation of the inner hair cells in residually hearing animals^12,13^.

The development of an optoacoustic stimulation was advanced by e.g. Wenzel et al. in hearing aid applications and for inner ear stimulation by Kallweit et al.^12,14^. In a recent study, our group demonstrated the laser-induced tone generation by nonlinear optical breakdown in water as well as in a gel volume^13^. In this study the same technique is applied for sound generation in human inner ear specimens. To achieve an optical breakdown in a medium, the laser must fulfill the constraints of stress and thermal confinement, i. e. the laser pulse duration and the laser focus are small enough so that the local laser intensity is high enough. If this is fulfilled, a plasma with a high density of free electrons in a small volume around the laser focus is generated. This process produces a shockwave. The concatenation of laser pulses with varying repetition rates results in a train of acoustic clicks, such that continuous tones are created. In principle, this mechanism can be employed for arbitrary signals^15–24^.

This optoacoustic method can be potentially combined with electrical stimulation by cochlear implants for EAS applications to cover low frequencies or with higher pulse repetition rates the entire human hearing range^13^. In our present work, we used an integral pulse density modulation (IPDM) as opposed to pulse density modulation (PDM) from our previous study^13^ and translated the application to a fiber-optical setup to stimulate the round window in human temporal bones (TBs) acoustically. The stimulation was produced by modulated sequences of optoacoustically evoked clicks in a gel volume in direct contact with the round window of the cochlea. This study investigates the generation of acoustic tones between 20 Hz and 1 kHz. To achieve this, we conducted measurements with IPDM laser pulse patterns in three human cadaveric TBs and determined the equivalent sound pressure level at the tympanic membrane by measuring the intracochlear pressure difference (ICPD) response in the cochlea across the basilar membrane.

## Methods

### Laser setup

In this paper, acoustic signals were produced by the nonlinear optoacoustic effect inside a viscous medium in contact with the round window. To achieve an optical breakdown^20^, a nanosecond pulsed near infrared (NIR) laser system (HELIOS 1064-5-50, Coherent, Inc.) with 1064 nm wavelength, a pulse duration of 0.7 ns and a maximum pulse energy of 100 µJ was used. Our setup used in a previous publication^13^ was modified in order to place the focus of the laser pulses inside a gel volume in direct contact to the round window membrane^24^. By the nonlinear optoacoustic effect, sequences of pressure waves were generated inside the gel and transmitted acoustically into the cochlear fluid. This layout is able to produce an acoustic stimulus, while keeping the optical breakdown and its unwanted side effects like e.g. potential tissue damage outside the cochlea^25^.

**Figure 1:**
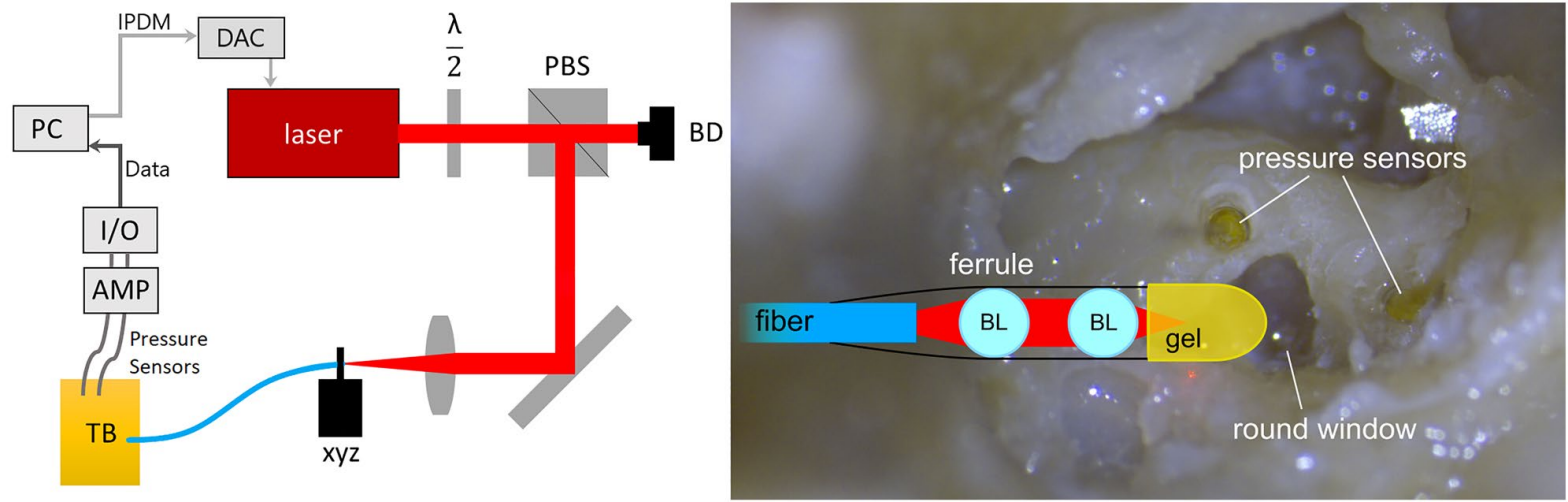
Left: Laser setup with half wave plate (λ/2), polarizing beam splitter (PBS), beam dump (BD), three-axis translation stage (xyz), temporal bone (TB) in which the two pressure sensors were placed inside the scala vestibuli and scala tympani, connection to the signal conditioner (AMP), to the input/output device (I/O) and to the computer (PC). The computer also creates the modulated signal (IPDM) as a digital pulse train that is passed onto a digital-analog converter (DAC). The analog signal controls the laser. Right: End of fiber with two ball lenses (BL) glued into a ferrule with optical adhesive and gel placed at the laser focus site. The tip of the gel is in direct contact with the round window membrane of the cochlea.

The focusing setup at the fiber end was inspired by the publication of Yang, Guang et al.^26^, and was previously used by our group to investigate cavitation dynamics in confined volumes^24^. The laser was coupled into an optical fiber (step-index multimode fiber, FP1000ERT, numerical aperture (NA) = 0.5, core diameter = 1 mm, Thorlabs UK) (Fig. 1 top) and focused at the outlet by two ball lenses (43709, Lens ball, Edmund optics, Inc., US, diameter = 1.5 mm, refractive index = 1.517). For further technical details see^24^. The beam was focused into a cylindrical viscous gel volume of approximately 3 mm in diameter and 4 mm in length made of PNC 400 gel in distilled water at a concentration of 10 g/l (Fig. 1 bottom). This gel plug was positioned in the round window niche and the laser fiber was placed such that the fiber tip aimed towards the direction of the round window. To adjust the laser power, a half wave plate and a polarizing beam splitter were placed in the beam path.

The resulting NA of the setup with the ball lenses was calculated with Zemax with a focus in water and a 70%-ratio of the diameter of the laser beam to the ball lens diameter to be NA = 0.6. The calculated ellipsoidal axes of the laser focus were approximately 1.8 µm × 570 µm with a threshold energy for an optical breakdown in water of ca. 2 µJ at a pulse length of 0.7 ns, which was calculated with a computer model of the free electron density^21^.

### Integral pulse density modulation

The integral pulse density modulation (IPDM) of the laser pulses is inspired by Li and Jones^27^. It is a further development from an earlier pulse density modulation (PDM) method that was already used in a previous study of our group^13,28^. In both methods, the uniform laser pulses can be spaced with varying time gaps between pulses to modulate an arbitrary signal. To generate pure tones here, we chose a periodically repeating sine wave as modulation. The previous PDM introduced a 180° phase delay to the initial signal, meaning that the pulse density was highest, when the signal amplitude was lowest. Also, the new modulation accurately scans the whole signal in contrary to the old PDM that compares the signal to a linearly rising saw-tooth and generates a pulse at each intersection^13^.

**Figure 2:**
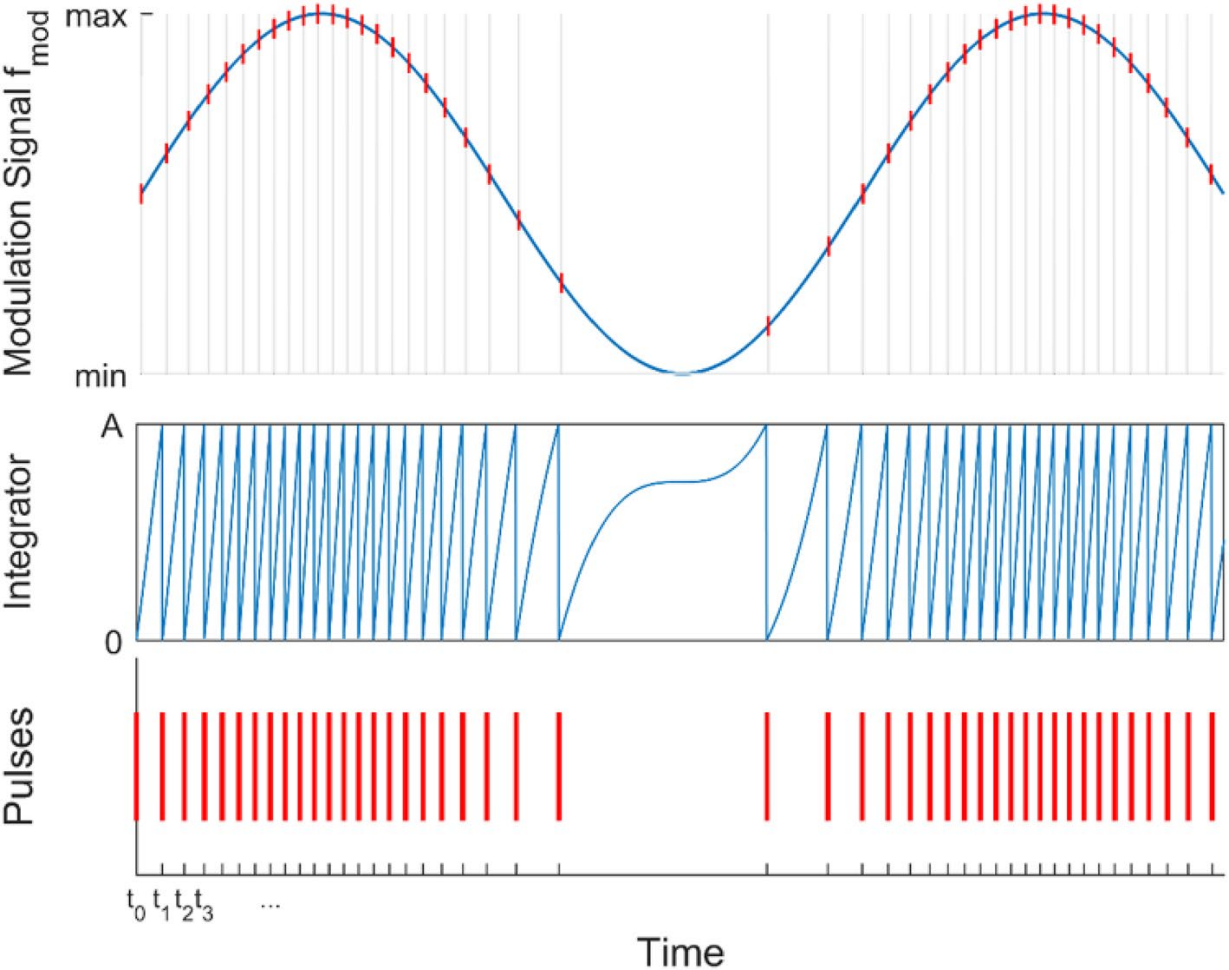
Top: Illustration of the integral pulse density modulation with the modulated sine wave signal. The red/grey lines indicate the pulse timings. Middle: Illustration of the integral over the sine wave signal. Once the threshold A is reached, a laser pulse will be fired and the integral is reset. Bottom: The resulting pulse sequence.

For the IPDM, the trigger for the next pulse is determined by integrating over the modulation signal with a constant chosen threshold^27^. When the integral of the modulation exceeds the threshold, a pulse is triggered and the integration starts over from zero at this point. The integral was approximated by numerical forward integration (Fig. 2) for a discrete time scale of 1 µs, adapted to the sampling rate of the laser controller being 1 MHz. The laser can be triggered at an arbitrary rate of up to 20 kHz, hence the maximum pulse repetition rate (PRR) for the modulation had to be capped at 50 µs. Furthermore, a constant minimum of 10,000 pulses per second was produced by the modulation for all the generated acoustic frequencies. This was chosen for historical and technical reasons. This ensured a minimum of at least 10 pulses per period of the highest modulation frequency tested (1 kHz). Considering these limitations, a binary array representing the pulse sequence to control the laser pulse timing was created. It is important to note that, whereas the gap between two pulses varied due to the modulation, the individual pulse energy and duration of each laser pulse remained constant.

### Pressure measurements and ICPD calculation

The pressure measurements in the cochlea were performed with fiber-optical sensors in the scala tympani and scala vestibuli as demonstrated by Grossöhmichen et al.^29^. Three TBs were prepared by surgeons with an access to the round window niche and to the middle ear ossicles. Human cadaveric temporal bones were obtained from the anatomy department from donors who had given their written informed consent. For experiments, TBs were harvested < 48 h post mortem and stored for later use at − 21 °C. Preparations and experiments were performed within 12 h after thawing at room temperature with the TBs being periodically rinsed with saline to prevent mechanical changes by drying. An experienced surgeon performed an extended mastoidectomy with a posterior tympanotomy. The facial nerve was dissected for wide access to the round window niche and stapes footplate (SFP). Additionally, the round window (RW) was exposed by drilling the round niche overhang, leaving the bony rim and the RW membrane intact. All experiments and protocols were approved prior by the local ethics committee (Medical School Hannover 10495_BO_K_2022) and were carried out in accordance with relevant guidelines and regulations.

For experiments, the TB preparations were mounted in a laboratory clamp fixed to a magnetic stand on a vibration isolated table (LW3048B, Newport, Germany). In each specimen the middle ear transfer function (METF) was measured to confirm compliance with the ASTM standard (ASTM F2504–05 Standard Practice for Describing System Output of Implantable Middle Ear Hearing Devices (IMEHDs))^30,31^. Then two fiber-optic pressure sensors (FOP-M260 NS 1050C, FISO Technologies, Canada) were inserted through cochleostomies into scala vestibuli (SV) and scala tympani (ST) and fixed with glue. After placing the pressure sensors the METF and the ICPD in response to acoustic stimulation was measured as reference. The pressure sensor signal conditioner (Veloce 50, FISO Technologies, Canada) was connected to the input/output card (NI USB 6356, NI, National Instruments Corp.), which was connected to a computer to readout and save the data in Matlab (R2021a, Mathworks, USA). Pressure sensors were calibrated with a reference microphone (MV 302, Microtech Geffel, Germany) between 100 Hz to 10 kHz. Before the measurements, the gel volume was positioned in the round window niche with close contact to the round window membrane and the laser fiber was placed with contact to the gel at ~ 2 mm distance to the round window with a micro-stage. Laser pulse sequences with 12 different modulation frequencies (20 Hz, 60 Hz, 100 Hz–1 kHz in steps of 100 Hz) were applied and the intracochlear pressure responses were recorded @ 1 MHz sample rate for 96 s each. Due to the large data size, the entire signal was split into multiple sections with 50% overlap and a Hanning-Window was applied on each part before the fast Fourier transform (FFT) was performed. This approach reduces artifacts and, depending on the length of each part, makes it possible to obtain a desired frequency resolution of the resulting spectrum^32^. Afterwards, all sections (windows) were averaged to acquire the pressures that were created in both scalae. In further steps, the ICPD was calculated and, with the acoustic reference measurement at the beginning of the experiment, the obtained equivalent sound pressure levels (dB SPL) were determined. For the modulation frequencies of 20, 60, 300, 600, 700, 800, and 900 Hz the calibration was interpolated.

## Results

### Pressure measurements of laser-induced tone generation in temporal bone with IPDM

**Figure 3:**
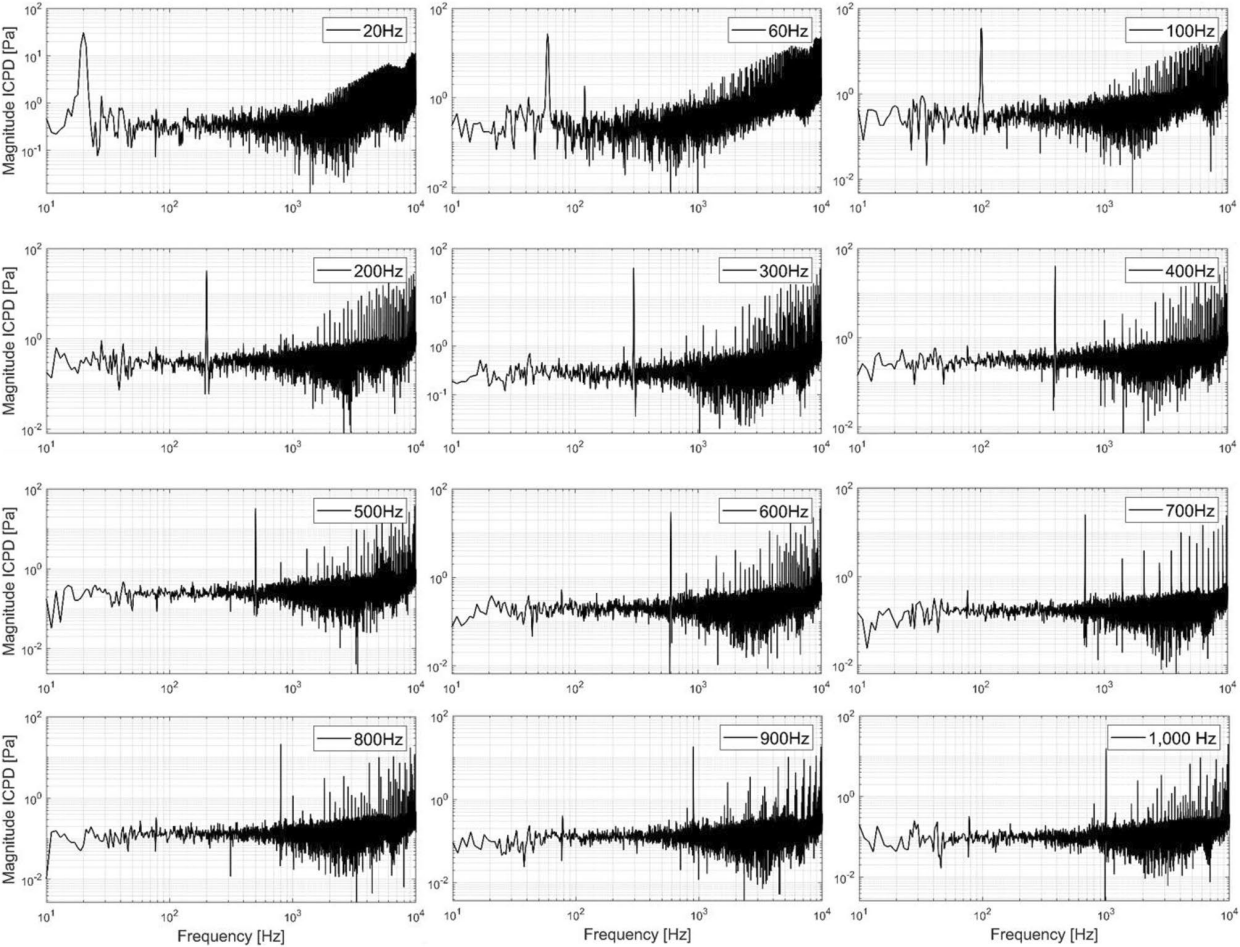
Frequency spectra of the intracochlear pressure difference (ICPD) of TB3 for all measured modulation frequencies. The spectra cover frequencies from 10 Hz to 10 kHz. The frequency resolution of the spectrum is 1 Hz per data bin. Measured pressures at each particular modulation frequency were far above noise-level.

**Figure 4:**
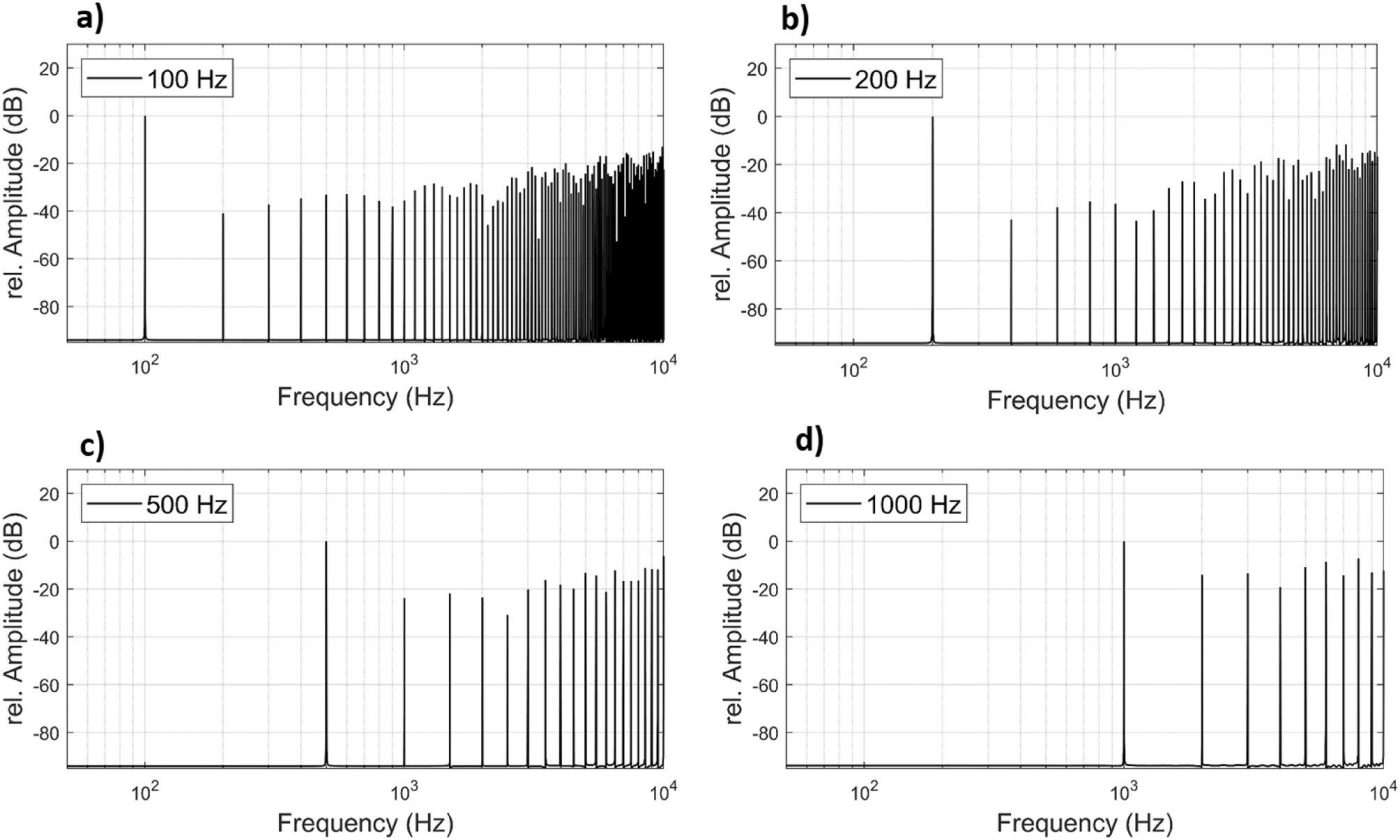
Simulated frequency spectrum of integral pulse density modulation for three example modulation frequencies of 100 Hz (**a**), 200 Hz (**b**), 500 Hz (**c**) and 1 kHz (**d**).

The frequency spectra of both sensors were calculated from raw recordings for all measurements of modulation frequencies between 20 Hz and 1 kHz via FFT. From these results, the ICPDs were calculated. The spectra obtained from the TB3 are displayed in Fig. 3. Results from TB1 and TB2 are displayed in Supplementary Figs. S1 and S2. The largest magnitude at low frequencies was far above noise level at the modulation frequency f_mod_ in all spectra. Additionally, distortions with increasing amplitudes towards higher acoustic frequencies were observed, starting from 2 to 3 kHz with TB3 having the most prominent distortions. Furthermore, for comparison with the measured results, we applied a FFT on the pulse sequence to obtain the theoretical frequency spectrum of the modulation (Fig. 4). It is apparent that, similar to the measurements, the main peak is located at the corresponding modulation frequency and equivalent distortions can be observed at higher acoustic frequencies.

**Figure 5:**
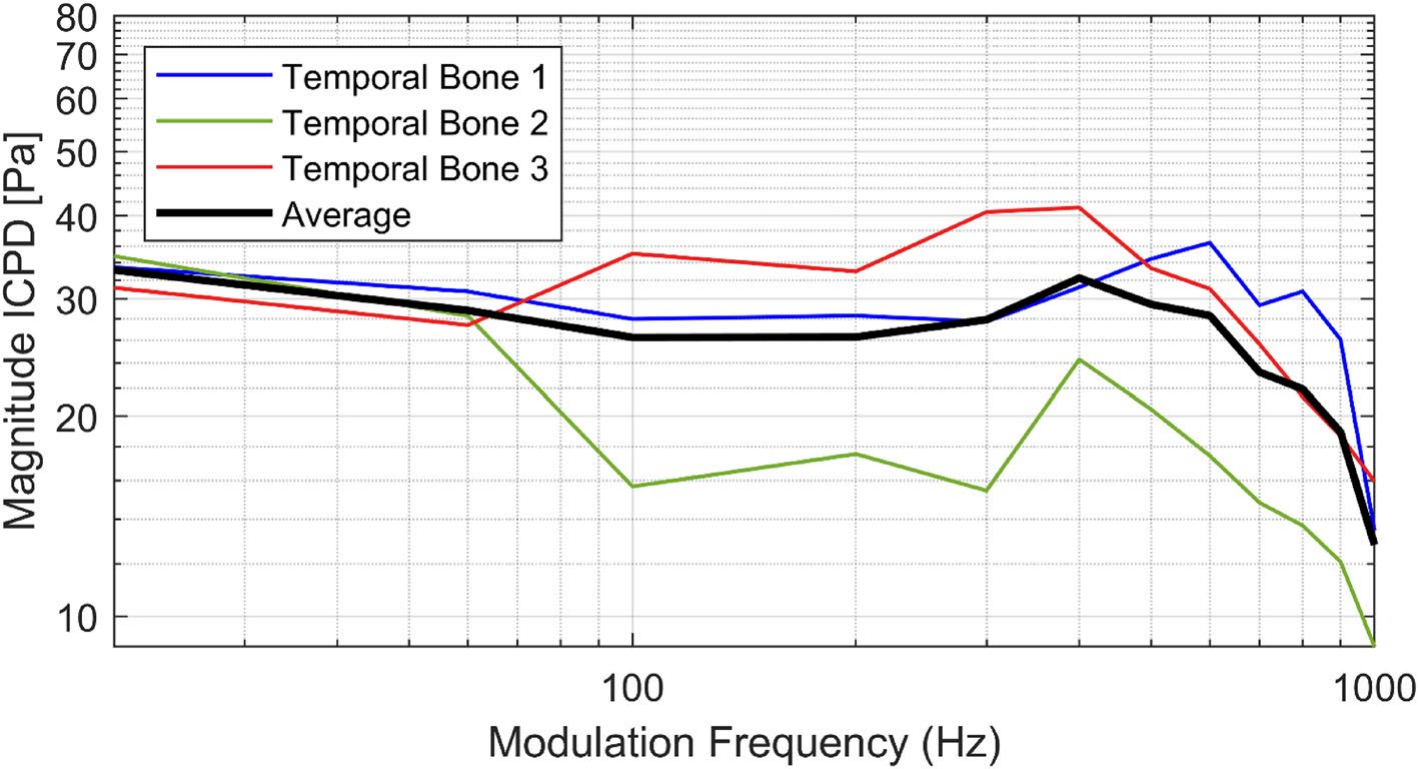
Measured intracochlear pressure difference between scala tympani and scala vestibuli for all investigated temporal bones (colored thin lines) at each modulation frequency (20–1000 Hz) and the average pressure (black thick line).

The resulting ICPDs of all modulation frequencies showed similar behavior for all TBs (cf. Fig. 5). In the first and third specimen similar pressure values were measured with a deviation of < 10 Pa across all modulation frequencies whereas in the second specimen lower pressures were recorded. At low modulation frequencies the pressures were highest and decreased to 9–14 Pa at 1 kHz.

### Equivalent sound pressure level output

**Figure 6:**
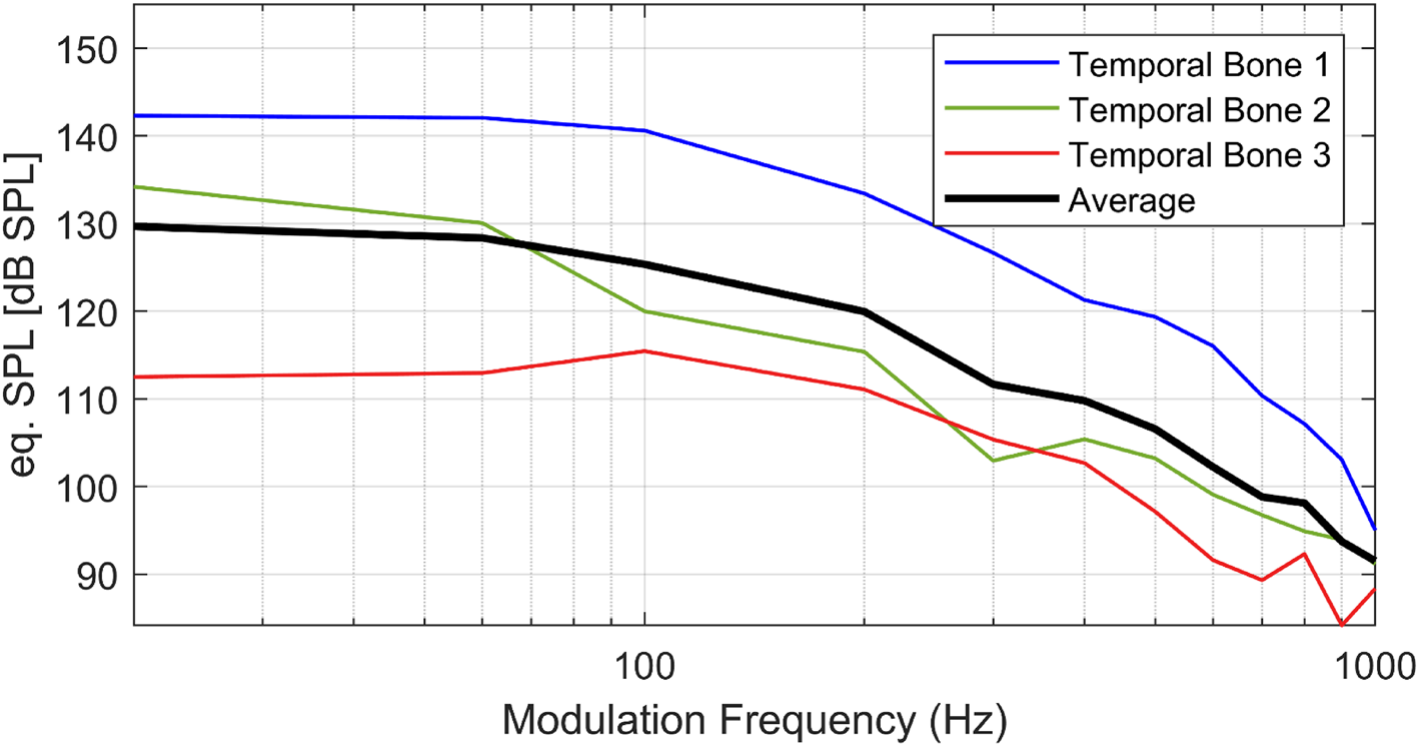
Equivalent sound pressure level at the tympanic membrane obtained by intracochlear pressure measurements in each temporal bones 1–3 (colored thin lines) and the average eq. dB SPL (black thick line).

To determine the equivalent sound pressure level (eq. dB SPL) of the optoacoustic stimulation at the round window membrane the equivalent sound pressure output was determined using the ICPD and the response to acoustic stimulation. Grossöhmichen et al. have demonstrated the feasibility of this approach and that results from temporal bones coincide with clinical results^33^. Optoacoustically generated acoustic responses were 110–140 eq. dB SPL at low modulation frequencies ≤ 200 Hz that decrease to roughly 90 eq. dB SPL at 1 kHz (Fig. 6).

## Discussion

### Integral pulse density modulation

In our study, we showed that laser-induced tone generation with the nonlinear optoacoustic effect and an integral pulse density modulation technique can be successfully used in stimulating the human round window membrane. We were able to measure responses in ICPD for all modulation frequencies between 20 Hz and 1 kHz. We employed an IPDM with pulse densities between the maximum repetition rate of our laser of 20 kHz and an a priori set minimum pulse rate of 10 kHz for all modulation frequencies. Furthermore, we compared the measured pressure spectra with the theoretical IPDM spectra that can be achieved with the parameters of our laser (cf. Figs. 3 and 4). It was possible to show, that our setup achieves a response that behaves similarly to the theoretical predictions where a high peak amplitude is present at the modulation frequency and smaller peaks at higher frequencies that correspond to unwanted distortion. The IPDM worked as expected and produced a measurable acoustic signal far above noise floor at the targeted modulation frequencies. While we did not test the modulation for frequencies > 1 kHz due to our limited pulse repetition rate, higher pitched tones can possibly be generated using the same method, if a laser with a sufficiently high pulse repetition rate is used.

### Optoacoustic stimulation of human inner ear

We were able to optoacoustically generate acoustic tone stimuli in the inner ear with fundamental frequencies ranging from 20 Hz to 1 kHz. We were able to generate equivalent sound pressure levels of 90–140 eq. dB SPL at frequencies ≤ 1 kHz in all of the three tested temporal bones. Eq. SPLs were highest at frequencies < 200 Hz and decreased towards higher modulation frequencies to an equivalent SPL of ~ 90 dB SPL at 1 kHz. Hence, audible signals were created at levels that surpass the dynamic range of severely impaired patients up to an uncomfortable level and even can be heard by people with profound (> 80 dB) hearing loss^34^ for all investigated frequencies from 20 Hz to 1 kHz. The decreasing performance towards high frequencies could be explained by the gel, as the viscosity of the medium increases the attenuation of sound waves of higher frequencies^13,24,35^. Although we could demonstrate the feasibility of sufficiently high sound pressure levels, hearing aid applications would require the coverage of the residual dynamic range between the (impaired) threshold and the uncomfortable level. This was not possible with the current limitations of the setup, i.e. the limited repetition rate. Here, higher repetition rates, in combination with an overall attenuation are required and may extend the usable dynamic range.

The eq. SPL of the ICPD was very similar between temporal bones 2 and 3, while the first TB showed an overall higher response of more than 20 dB at low frequencies (Fig. 6). However, towards the highest tested modulation frequency the difference was much smaller. The TB samples were tested, according to the ASTM protocol, before the optoacoustic stimulation was performed. Although, the variability in output levels in TB experiments is usually not negligible, additional sources of variability may have contributed. We suspect that the placement of the stimulation implant at the round window membrane needs to be improved. Parameters like the exact position of the implant or the initial pressure on the membrane caused by the contact might influence the performance. Finding ways to improve the consistency is therefore necessary.

### Future of optoacoustic stimulation using IPDM

**Figure 7:**
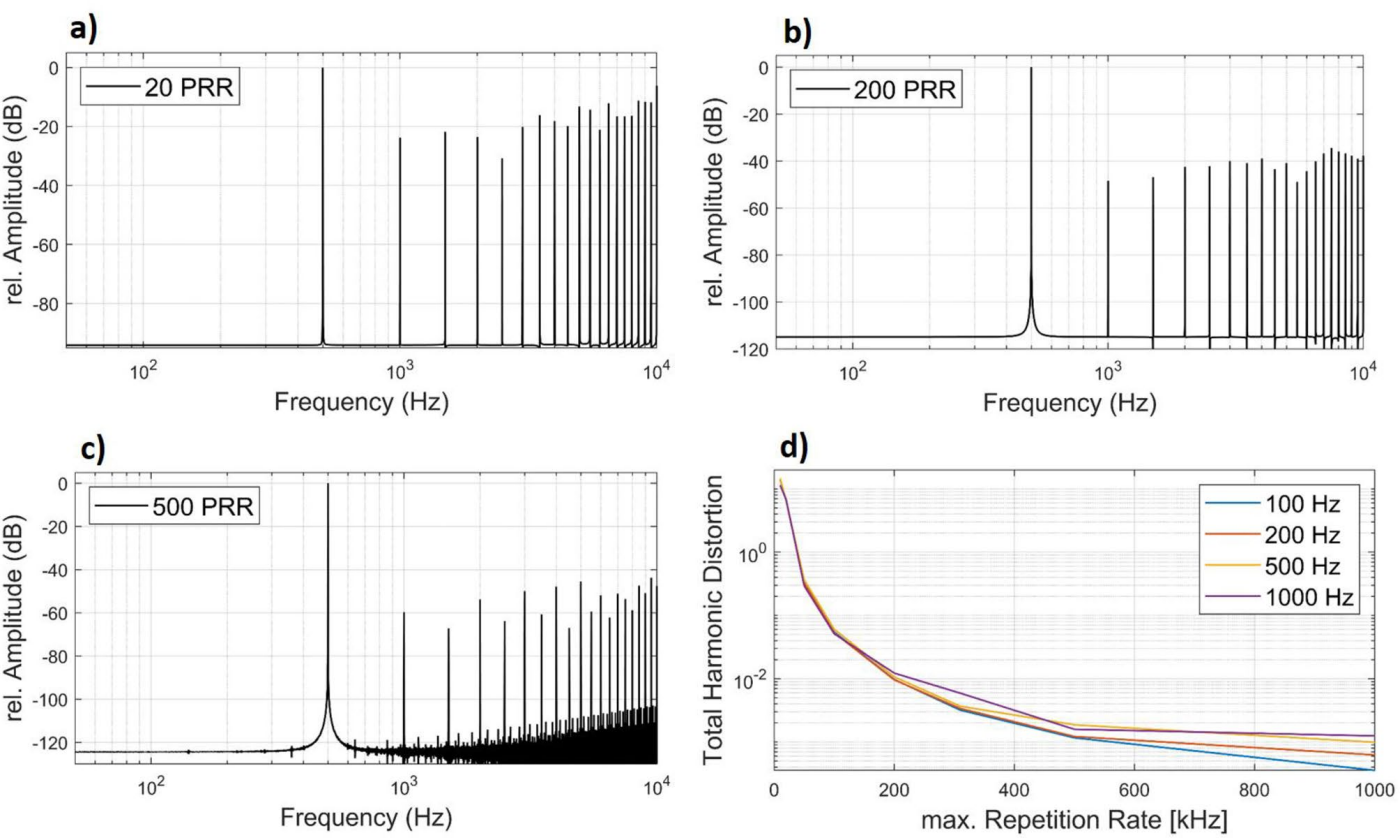
Simulation of the frequency spectrum of the IPDM signal at 500 Hz created by our algorithm at a pulse repetition rate (PRR) of 20 kHz (**a**), 200 kHz (**b**) and 500 kHz (**c**). (**d**) The resulting total harmonic distortion depending on the max. repetition rate is shown for four different modulation frequencies ranging from 100 Hz to 1 kHz.

The frequency spectra of the IPDM simulation showed that the attenuation of the harmonic distortion depends on the possible maximum pulse frequency. The laser in use achieved a maximum pulse rate of 20 kHz and we investigated the modulation with higher laser repetition rates in a simulation. We calculated the total harmonic distortion (THD) of the simulation for all harmonic frequencies ≤ 20 kHz. The THD is attenuated greatly when the maximum pulse repetition rate is increased (Fig. 7). The simulation shows that even with maximum repetition rates as low as 500 kHz, THDs of 0.12–0.19% in acoustic output can be achieved for modulation frequencies ≤ 1 kHz. For high modulation frequencies of up to 10 kHz, a THD of < 1% can be achieved by a repetition rate of at least 200 kHz.

The IPDM used in this paper builds a strong foundation for future studies and appears to be a viable solution to generate arbitrary acoustic signals. The precise frequency generation and the low distortion might be an indicator that a hypothetical implant using this method may be able to produce clear acoustic signals if sufficiently high repetition rates are used. Although 500 kHz as maximum repetition rate appears sufficient for EAS applications that are characterized by residual hearing at low frequencies < 1.5 kHz and profound deafness above, which would further attenuate the perception of higher harmonics, hearing aid applications covering the entire audible frequency range are feasible. Higher repetition rates will enable low distortion and sufficient maximum output in the entire frequency range relevant for hearing aids (< 10 kHz), improving speech perception and providing adequate quality of music. Furthermore, higher repetition rates are required for an increased amplitude resolution allowing for an extended output dynamic range. A better performance of the current setup might be achieved by changing the viscous medium to attenuate the high frequency distortions even further. On the path towards a laser-based hearing implant, a miniaturized laser system is necessary to achieve a practicable size of the stimulation setup. Therefore, laser diodes with a sufficiently short pulse duration and high pulse energy are a promising approach. It was successfully shown that a laser diode that only meets the condition for thermal confinement was able to produce acoustic events^13^.

## Conclusion

The feasibility of low-frequency tone generation by laser stimulation with an integral pulse density modulation for modulation frequencies between 20 Hz and 1 kHz was demonstrated in three human cadaveric temporal bones. Optoacoustic stimulation yielded sufficient acoustic output for electro-acoustic stimulation applications at frequencies ≤ 1 kHz. By using an optical breakdown in a gel volume that was in contact with the round window membrane, audible signals were generated and measured by differential intra-cochlear pressure sensors in both scalae of the cochlea. The results reveal an output performance of 110–140 eq. dB SPL at low modulation frequencies and up to 90 eq. dB SPL at 1 kHz in each of the three temporal bones. This method is therefore potentially able to produce sufficiently loud arbitrary acoustic output for patients requiring electro-acoustic-stimulation.

### Supplementary Information

Supplementary Figures.

## Data Availability

The data that has been used is available from the corresponding author upon request. E-mail: maier.hannes@mh-hannover.de.
