# Supplementary material for: Acoustic stimulation of the human round window by laser-induced nonlinear optoacoustics
Source: Sci Rep. 2024 Apr 8;14:8214. doi: 10.1038/s41598-024-58129-0 (PMC11001906; doi:10.1038/s41598-024-58129-0)
Supplement: Supplementary file 1 — Supplementary Figures. [file 41598_2024_58129_MOESM1_ESM.docx]

**Supplementary Figures**


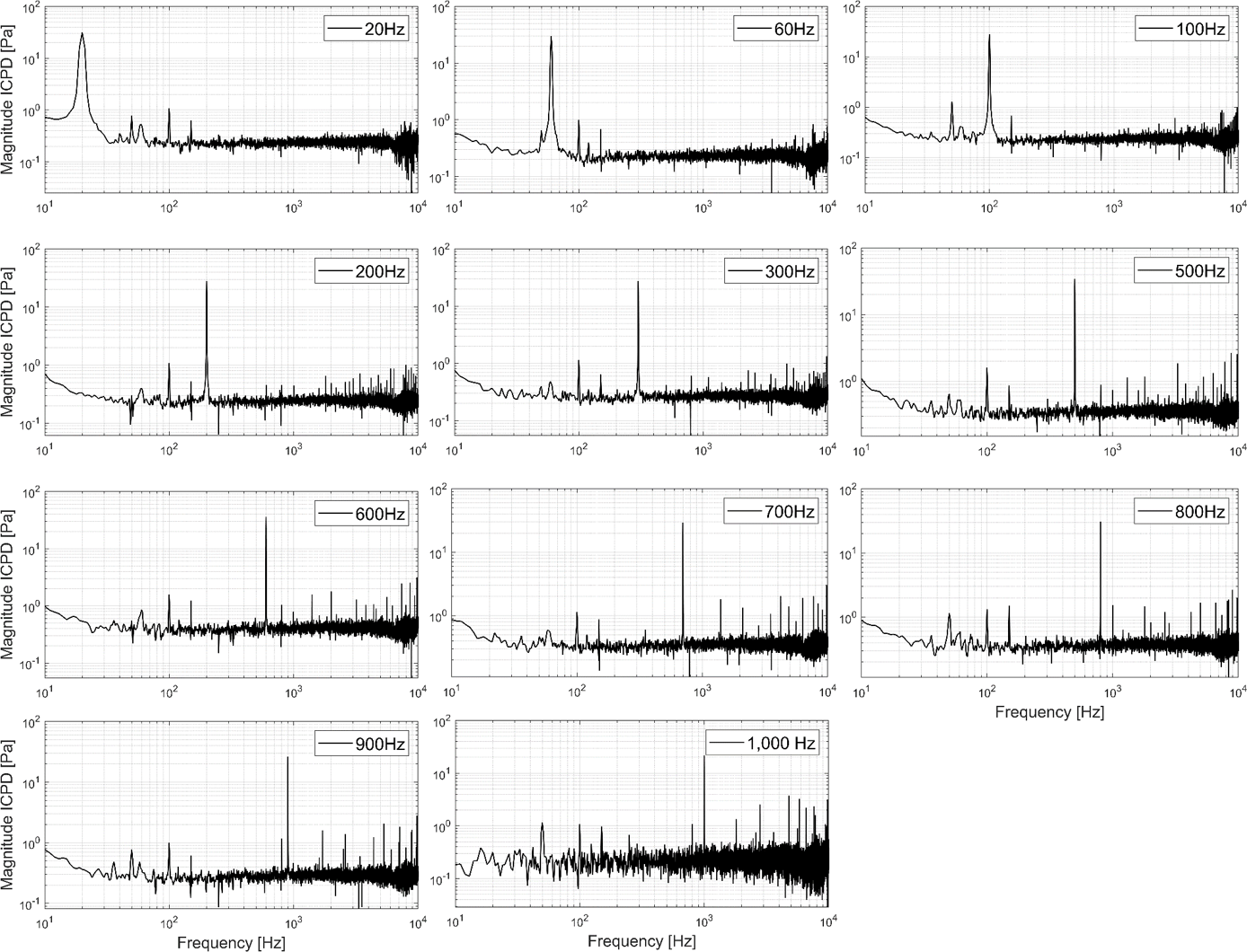


Fig. S1: Frequency spectra of the intracochlear pressure difference (ICPD) for all measured modulation frequencies for temporal bone 1. The frequency resolution is 1 Hz per data bin. Measured pressures were far above noise-level at each particular modulation frequency.


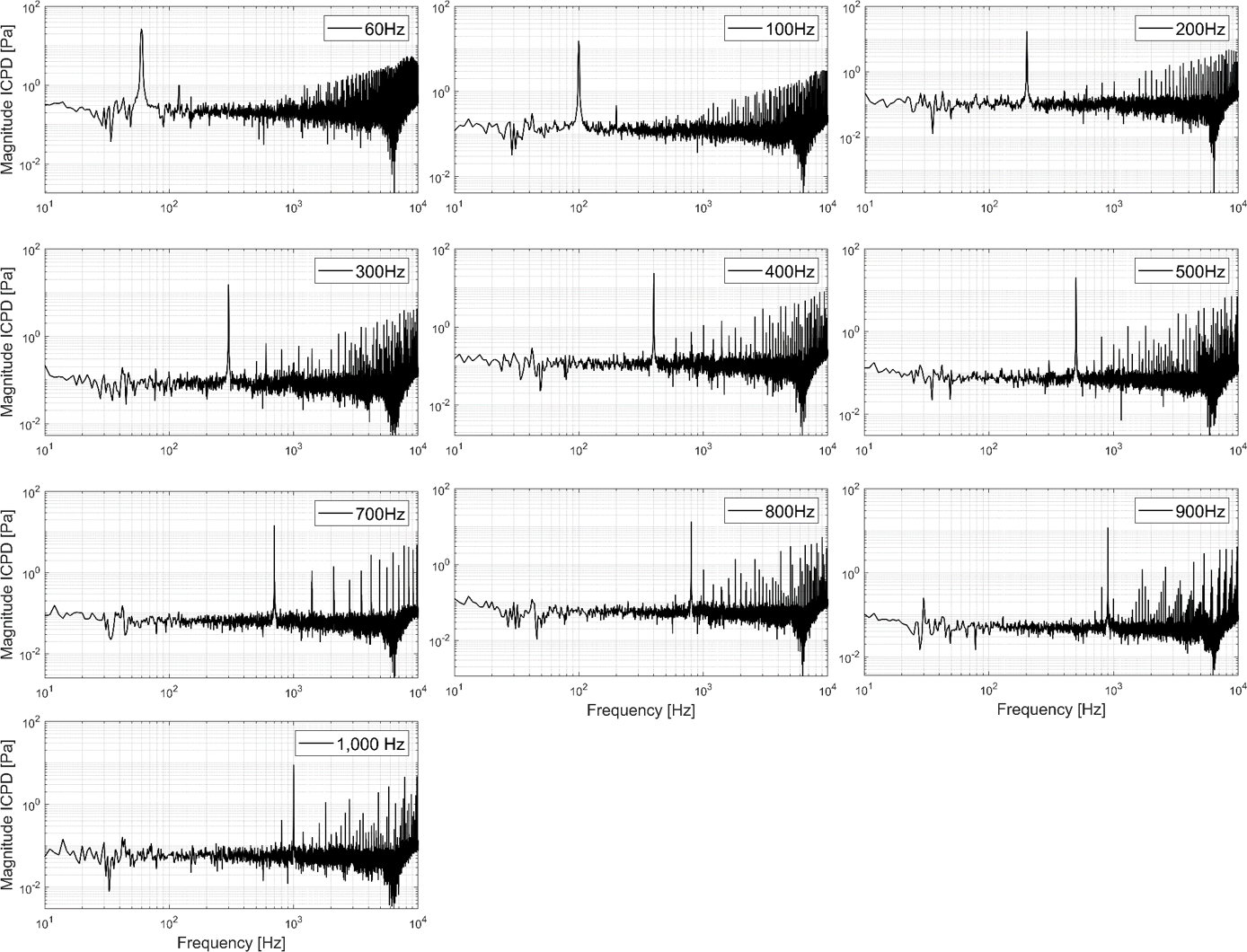


Fig. S2: Frequency spectra of the intracochlear pressure difference (ICPD) for all measured modulation frequencies for temporal bone 2. The frequency resolution is 1 Hz per data bin. Measured pressures were far above noise-level at each particular modulation frequency.
